# General practice follow-up after hospital discharge in older adults: retrospective record analysis in UK primary care

**DOI:** 10.3399/BJGP.2025.0627

**Published:** 2026-06-30

**Authors:** Naomi Klepacz, Annabelle Long, Zakia Shariff, Simon EF Spencer, Hema Mistry, Frances Griffiths, Jeremy Dale, Rachel A Spencer

**Affiliations:** 1 Warwick Applied Health, University of Warwick, Coventry, UK; 2 Department of Health and Community Sciences, University of Exeter Medical School, University of Exeter, Exeter, UK; 3 Department of Statistics, University of Warwick, Coventry, UK; 4 Warwick Clinical Trials Unit, University of Warwick, Coventry, UK; 5 Warwick Medical School, University of Warwick, Coventry, UK

**Keywords:** care utilisation, general practice, patient discharge, patient readmission, error, harm

## Abstract

**Background:**

Most hospital admissions are for older patients, who are more likely to be medically complex. Primary care risks after hospital discharge and readmission trajectories are relatively unexplored.

**Aim:**

To estimate the frequency and nature of errors and harms linked to discharge summary processing in general practice for patients aged ≥65 years; and to explore associations with readmission and healthcare utilisation, using a novel primary care data source.

**Design and setting:**

Retrospective cohort study using electronic health records (EHRs) from seven purposively sampled general practices in the West Midlands, UK.

**Method:**

EHRs of patients aged ≥65 years discharged between October 2022 and October 2023 were reviewed. Outcomes included post-discharge healthcare utilisation and costs, frequency and type of discharge actions, error rates (failures to complete requested actions), harms (severity, attribution, and preventability), and 90-day readmissions. Multivariable logistic regression identified predictors of errors, harms, and readmission.

**Results:**

Within the cohort of 263 discharged patients, 186 (70.7%) accessed post-discharge care; with 47 out of the 263 patients (17.5%; 95% confidence interval = 13.7 to 23.0) having related readmissions within 90 days. In total, 551 actions were requested in 160 discharge summaries. For those patients who required an action, 20.6% experienced error and 4.4% experienced harm. Most harms resulted in hospital readmission and three out of eight were preventable in primary care. Error and harm disproportionately affected patients who had dementia or a recorded carer.

**Conclusion:**

General practice should review their processes for responding to patient discharge information in order to improve patient safety post-discharge. Further research into tools to assist practices with transitions is warranted.

## How this fits in

Older patients frequently use general practice post-discharge care at point of need. Older patients (particularly those with carers or a dementia diagnosis) are at risk of harm from post-discharge primary care. Error rates in processing hospital discharge summaries in general practice appear to be improving in the last decade but harm remains. Recognition of a funded general practice post-discharge care episode and research into interventions for this cohort are crucial given the emphasis on the shift from secondary to primary care and the UK ageing population.

## Introduction

Transition from hospital to home is a recognised point of vulnerability for older adults with multimorbidity, frailty, and polypharmacy.^1,2^ In England, people aged ≥65 years account for >6 million hospital admissions annually, including 2.6 million emergency admissions.^3^ While national guidance emphasises safe discharge and coordinated follow-up,^4,5^ discharge transitions remain a persistent risk,^6^ repeatedly featuring in patient concerns.^7^ In this article, the authors revisit the epidemiology of discharge-related harm in primary care in a 10-year update to their prior work.^1^

Discharge summaries are frequently a source of communication failure between hospital and general practice: often delayed, unclear, or missing key clinical details making safe and timely care difficult.^8–10^ The authors’ prior work found that nearly half of discharged older patients’ summaries requiring general practice action were not fully processed, and 8% were linked to moderate or severe patient harm.^1^ Patients and carers also report feeling unprepared for discharge and unsure who to contact once home, leaving general practice to manage unresolved issues.^7^

Though hospital-to-home transitions occur within a wider system of community and out-of-hospital services, general practice provides the ongoing, longitudinal follow-up for most older adults after discharge. Despite this, most research has focused on hospital-led (‘transitional care’) interventions to reduce readmissions.^11^ Far less is known about how post-discharge care is organised within general practice; as a result, few interventions exist.^12^ Recent ethnographic work describes wide variation in discharge summary handling, reflecting local workflows and staffing rather than shared protocols.^13^ Increasing frailty and ‘left shift’^14^ of activity from hospital to primary care place an unknown burden on general practice in the post-discharge space.

Medication reconciliation is a particular risk, with 15% of older adults experiencing medication-related harm within 8 weeks of discharge, much of it preventable and often leading to further healthcare use.^15^ Interventions that reduce readmissions^16^ are not routinely integrated into general practice.

This study aimed to:

- describe patterns of (primary and urgent) care utilisation following discharge in patients aged ≥65 years, including economic analysis;
- explore the relationship between primary care follow-up and hospital readmission, including the feasibility of a novel use of primary care electronic health records (EHRs) data to examine post-discharge outcomes; and
- identify general practice-originated errors and harms associated with discharge.

This study provides a 10-year update to the authors’ previous retrospective records analysis of discharge-related harm in general practice.^1^

## Method

### Study design

A retrospective cohort study using anonymised EHRs from patients registered at seven general practices in the West Midlands, UK. Practices were purposively selected from those expressing interest to provide maximum variation in list size, rural/urban setting, ethnic diversity, and socioeconomic profile (based on practice-level Index of Multiple Deprivation [IMD] data) (Table 1).

**Table 1.** Study practice characteristics

Practice study code^a^	Practice list size, *n* ^b^	Patient population aged ≥65 years, %^c^	Ethnicity, White, %^c^	Patients per WTE GP^c^	IMD decile^c^	Setting^c^	CQC rating^d^
10	12 300	14.8	74.1	1065	2	Urban	Good
11	21 000	15.4	68.7	1646	2	Urban	Outstanding
13	26 500	5.6	57.3	2470	2	Urban	Good
14	8500	18.7	83.3	1758	3	Urban	Good
16	12 400	26.8	91.9	883	10	Semi-rural	Outstanding
17	8000	19.2	88.8	909	8	Rural	Good
18	5700	30.0	93.4	1145	8	Rural	Good

^a^Practice study codes correspond to those used in authors’ ethnographic study,^13^ missing numbers occur where re-recruitment to this stage of the study failed. ^b^Data from practice records, practice list sizes rounded to nearest 100 to preserve anonymity. ^c^2025 data from General Practice Workforce.^32^ ^d^Data from CQC website.^33^ CQC = Care Quality Commission. IMD = Index of Multiple Deprivation. WTE = whole-time equivalent.

### Record inclusion criteria

Records from all patients aged ≥65 years discharged from hospital from October 2022 to October 2023 were included. Patients with <90 days of EHR data post-discharge were excluded.

### Data collection

The third and last authors, an academic pharmacist and an academic GP, respectively, reviewed EHRs onsite at each practice. The first six post-discharge contacts in general practice were extracted in chronological order. Data quality was ensured by working as a dyad with live discussion between the clinician reviewers to prevent misclassification of failures. Eligible records were identified using local search tools, anonymised at the point of extraction, and entered into a structured Microsoft Excel case report form (Box 1). Methods were aligned with those of the authors’ previous study^1^ to allow comparison.

**Box 1.** Variables extracted from electronic health records, by domain

Domain	Variable
Patient characteristics	Age, sex, ethnicity, dementia diagnosis, and carer status
Hospital admission details	Dates of admission and discharge, admitting hospital, admission type (elective or emergency), hospital department, primary discharge diagnosis, and length of stay
Urgent and emergency care utilisation	Readmission dates, accident and emergency (A&E) attendances, ambulance callouts, walk-in centre (WIC) use, and out-of-hours (OOH) contacts
General practice utilisation	Up to six related contacts post-discharge, including date, clinician role (for example, GP, nurse, and pharmacist), and mode (face-to-face, telephone, SMS [Short Message Service], and home visit)
Medicines reconciliation actions^a^	Medication changes post-discharge (newly prescribed, stopped, and dose-adjusted repeats)
Follow-up actions^a^	Any tests ordered, referrals initiated, or other documented requests linked to the discharge event
Safety outcomes	Presence of errors (single or multiple), associated harm, severity and preventability of harm, attribution of harm to error, and whether hospital readmission resulted from the error (including length of stay)

a *An ‘action’ is a request made by the discharge summary author of the general practice team*.

### Data analysis

Descriptive statistics summarised general practice and patient characteristics, post-discharge care use, and discharge actions. Frequencies and proportions were reported for categorical variables; means and standard deviations (SDs) (or medians and interquartile ranges [IQRs]) for continuous data. Multivariable logistic regression identified predictors of: failure to complete discharge summary actions (errors); harms resulting from these errors; and readmission within 90 days. Variables were selected for clinical relevance and compared to the authors’ 2014 data.^1^ Odds ratios (ORs), 95% confidence intervals (CIs), and *P*-values were reported. The likelihood ratio test assessed overall significance of categorical predictors. Subgroup analyses examined outcomes by age group and admission type. Analyses were conducted using R statistical software. Healthcare service use was costed descriptively using standard NHS reference sources.^17–19^ Mean and total costs were reported by service type and patient subgroup. Harm severity was graded by the clinical academic reviewers using established World Health Organization scales,^17^ preventability was graded according to previously used scales.^1,18,19^

## Results

### General practice and patient characteristics

In total, seven general practices participated, with list sizes ranging from 5700 to 26 500 patients (Table 1). Patients aged ≥65 years comprised 5.6%–30.0% of practice populations. Practices varied in socioeconomic deprivation, ethnicity, rurality, and Care Quality Commission (CQC) rating. The ratio of patients to whole-time equivalent GPs ranged from 1:883 to 1:2470.

The cohort comprised 263 patients with a mean age of 77.4 years; 140 were male (53.2%). Thirty-three (12.5%) had a dementia diagnosis, 74 (28.1%) a recorded carer, 19 (7.2%) lived in a care home, and 16 (6.1%) had died within 90 days of discharge. Of the 263 patients, 150 (57.0%) had medical admissions and 113 (43.0%) had surgical admissions. Emergency admissions accounted for 184 cases (70.0%), comprising 98.7% of medical and 31.8% of surgical admissions. Mean hospital stay was 9.6 days (range 1–689 days), median 3 days (IQR 1–8 days) (data not shown).

### Post-discharge care utilisation

Within 90 days of discharge, 186 patients (70.7%) used healthcare services because of their index admission ‘attributable care’ (broken down by provider in Table 2). Total cost of attributable urgent and emergency care utilisation was £121 960.70; median cost of £681.00 per user (*n* = 52), mean £1451.91 (SD £2101.54). Total cost for attributable primary care was £13 821.66; median cost £72.79 per patient, mean £89.75 (SD £69.37) (data not shown).

**Table 2.** Post-discharge care utilisation (*N* = 263)

Category	Patients experiencing care,*n* (%)	Patients experiencing >1 episode, *n*	Total occurrences of care, *n*	Patients experiencing attributable care,*n* (%)	Total occurrences of attributable care, *n*
General practice	174 (66.2)	109^a^	n/a^b^	152 (57.8)	416
Hospital admission	63 (24.0)	15	80	46 (17.5)	54
Ambulance	50 (19.0)	1	52	43 (16.3)	43
A&E	49 (18.6)	2	52	38 (14.4)	40
OOH	26 (9.9)	0	26	25 (9.5)	25
WIC	1 (0.4)	0	1	EHR incomplete	n/a

^a^Mean number of appointments = 1.4 (SD 1.58). ^b^Where attributable contact occurred within 21 days, subsequent attributable contact data were collected across 3 months up to a maximum of six contacts per patient. A&E = accident and emergency. EHR = electronic health record. n/a = not applicable. OOH = out of hours. SD = standard deviation. WIC = walk-in centre.

General practice care was mostly offered on the telephone (48.6%; face-to-face, 38.0%; home visit, 9.4%; and SMS, 3.6%) by GPs (64.9%; nurse, 18.8%; pharmacist, 8.4%; allied health professionals, 4.1%; and non-clinical staff, 3.1%). Of the patients with attributable general practice care post-discharge, 120/152 (78.9%) had index emergency admissions. For 127/152 (83.6%) patients, their attributable general practice contact occurred within 14 days of discharge. No other services were used by 103/174 (59.2%) of the patients who were seen in general practice (data not shown).

Academic clinician record reviewers judged 47/263 (17.9%, 95% CI = 13.7 to 23.0) patients to have been directly attributably readmitted within 90 days of discharge, with 5/47 (10.6%) due to harms (data not shown). In univariate models (Table 3), dementia was associated with significantly increased odds of readmission (OR 2.69; 95% CI = 1.20 to 6.04; *P* = 0.016), as was a hospital stay of 5–14 days versus <5 days (OR 3.01; 95% CI = 1.52 to 5.99; *P* = 0.002) (analysis of variance: *P =* 0.007*)*.

**Table 3.** Modelling of errors, harms, and readmissions

Outcome and predictor	Odds ratio	95% CI	*P*-value
**Error**			
Carer on EHR	2.83	1.23 to 6.52	0.0143
Resident in care home	2.60	0.70 to 9.63	0.1526
Dementia diagnosis	4.59	1.74 to 12.05	0.0026
Patient sex, male	1.66	0.76 to 3.63	0.2005
Non-white ethnicity^a^	1.88	0.44 to 8.05	0.3949
Admission type, surgical	0.51	0.18 to 1.43	0.1987
GP contact post-discharge	0.86	0.36 to 2.05	0.7352
GP contact related to index admission	0.75	0.33 to 1.67	0.4777
Number of GP contacts	1.01	0.83 to 1.24	0.9116
GP contact within 1 week	0.94	0.43 to 2.05	0.8716
Seen by GP	0.99	0.46 to 2.14	0.9824
Length of stay, 5–14 days	2.02	0.84 to 4.86	0.1179
Length of stay, >14 days	1.85	0.62 to 5.45	0.2677
**Harm**			
Dementia diagnosis	5.63	1.16 to 27.19	0.0317
Patient age	1.06	0.96 to 1.18	0.2327
Patient sex, male	0.72	0.16 to 3.33	0.6754
Admission type, surgical	0.52	0.06 to 4.48	0.5539
GP contact post-discharge	2.05	0.24 to 17.59	0.5117
GP contact related to index admission	1.14	0.21 to 6.10	0.8758
Number of GP contacts	1.22	0.85 to 1.75	0.2703
GP contact within 1 week	2.01	0.43 to 9.30	0.3713
Seen by GP	0.89	0.19 to 4.11	0.8805
Length of stay, 5–14 days	5.88	0.67 to 51.78	0.1106
Length of stay, >14 days	2.39	0.14 to 39.61	0.5423
**Readmission**			
Dementia diagnosis	2.69	1.20 to 6.04	0.0160
Patient age, per year increase	1.04	0.99 to 1.09	0.0619
Non-white ethnicity^a^	0.31	0.04 to 2.39	0.2585
Emergency admission	1.50	0.72 to 3.13	0.2759
Patient sex, male	1.90	0.98 to 3.68	0.0561
Department of admission, surgical	0.98	0.52 to 1.85	0.9497
GP contact post-discharge	1.10	0.56 to 2.15	0.7887
GP contact related to index admission	1.09	0.58 to 2.08	0.7852
Number of GP contacts	1.08	0.92 to 1.27	0.3522
GP contact within 1 week	1.04	0.54 to 2.01	0.8981
Seen by GP	1.32	0.70 to 2.50	0.3934
Carer recorded on EHR	1.53	0.76 to 3.06	0.2309
Resident in care home	1.98	0.66 to 5.96	0.2256
Length of stay, 5–14 days	3.01	1.52 to 5.99	0.0016
Length of stay, >14 days	1.37	0.47 to 3.99	0.5637

^a^Reference category = ‘British’, ’British or Mixed British’, ’English’, ’Irish’, ’Other Mixed White’, ’Other White’, ’White’, ’White British’, and ’White Irish‘. Reference categories for each row can be found in Supplementary Information S1. EHR = electronic health record.

### Actions, failure to complete actions (error), and harms

#### Actions

Overall, 160 (60.8%; 95% CI = 54.8 to 66.6) discharge summaries requested ≥1 actions be undertaken by the general practice team, totalling 551 actionable requests. Of these discharge summaries, 128 required medication reconciliation, with 384 medication change requests (mean 1.46 requests per patient, SD 2.29), comprising: 266 requests (69.3%) to start a new repeat medication; 76 (19.8%) to discontinue; and 42 (10.9%) to change a dose. Of the 160 discharge summaries requesting actions, 45 (28.1%) requested a follow-up test or investigations, totalling 58 actionable requests (mean 1.30 per patient, SD 0.55). Referral requests appeared in 11 summaries (6.9%), and 71 (44.4%) contained other GP requests, totalling 98 actions (mean 0.37 per patient, SD 0.75) (data not shown).

#### Failure to complete actions (error)

In total, 50 errors (incomplete action without explanatory free text in the EHR) were identified. Most errors were medication-related (*n* = 30), mainly related to new repeat medication (*n* = 24/30) and rarely due to discontinuing (*n* = 2/30), changing doses (*n* = 2/30), or reviewing/monitoring medications (*n* = 2/30) (see Supplementary Table S1 for list of drugs not actioned). A total of 10 errors involved follow-up of investigations (*n* = 4/10 blood tests and *n* = 6/10 imaging/procedures). There were eight ‘other action’ errors: wound checks (*n* = 2/8); ReSPECT forms (*n* = 2/8); and physiological monitoring (*n* = 4/8), and two referral errors were identified. Reasons for non-completion most commonly arose from ‘clinical reasoning not documented’ actions, inappropriate or unclear requests, or missing information in discharge summaries (see Supplementary Table S2).

Of the 263 patients in the cohort, 160 had ≥1 actions. Of these patients, 33 experienced at least one error and 13 experienced multiple errors. The overall per patient error rate was 20.6% (95% CI = 15.0 to 27.6), rising to 21.5% (95% CI = 14.7 to 30.2) in patients aged ≥75 years (data not shown). Dementia (OR 4.59; 95% CI = 1.74 to 12.05; *P* = 0.002) and having a recorded carer (OR 2.83; 95% CI = 1.23 to 6.52; *P* = 0.014) were statistically significantly associated with error (Table 3). Care home residence increased odds (OR 2.60; 95% CI = 0.70 to 9.63), but not significantly (*P* = 0.153). Sex, ethnicity, number of GP contacts, and post-discharge follow-up were not significantly associated with error. Longer hospital stays were associated with higher odds (5–14 days, OR 2.02 and >14 days, OR 1.85), though not significantly. No errors occurred among elective admissions.

#### Harms

Of 50 identified errors, eight caused harm to seven patients (per patient harm rate 4.4%; 95% CI = 2.1 to 8.8). All harms occurred following emergency admissions and in patients with a recorded carer. Incidence rose to 6.5% (95% CI = 3.2 to 12.9) in patients aged ≥75 years. Of the seven harmed patients, five had directly attributable readmissions (mean stay 5.2 days; range 1–12) (data not shown). Five harms were medication related, and three test related (Table 4). Six of eight harms were graded ‘substantial’ (resulted in readmission for five patients); one ‘required intervention’ by a healthcare professional (HCP); and one was ‘potential harm’. Five harms were ‘potentially preventable’ and three ‘preventable and attributable to primary care’. Harms were significantly more likely in patients with dementia (OR 5.63; 95% CI = 1.16 to 27.19*; P* = 0.032) (Table 3).

**Table 4.** Harms

Error type	Cause	Description of harm	Severity^a^	Preventability^b^	Hospitalisation, days
Failure to action new repeat medication	Clinical reasoning not documented	Infective exacerbation of COPD	4	2	6
Failure to review existing medicines	Multiple discharge summaries	Constipation and UTI	3	2	0
Failure to action new repeat medication	Clinical reasoning not documented	Insufficient pain management	4	4	1
Failure to action new repeat medication	Clinical reasoning not documented	Constipation and urinary retention	4	4	2
Failure to action new repeat medication	Clinical reasoning not documented	Insufficient pain management	2	4	0
Failure to review bloods	Failed contact	Anaemia	4	2	12
Failure to conduct test	Hospital request unclear	Repeat infection	4	2	9
Failure to order imaging	Hospital booking action	LRTI	4	2	1

^a^Severity scores: 1 = potential; 2 = inconvenience; 3 = intervention required; and 4 = substantial (including hospitalisation). ^b^Preventability scores: 1 = not preventable or originated in secondary care; 2 = potentially preventable; 3 = preventable; and 4 = preventable and attributable to primary care. COPD = chronic obstructive pulmonary disease. LRTI = lower respiratory tract infection. UTI = urinary tract infection.

## Discussion

### Summary

This study conducted a retrospective analysis of EHRs to examine post-discharge healthcare use, follow-up, and readmissions in patients aged ≥65 years. Post-discharge healthcare utilisation was high: nearly three-quarters of patients re-entered care within 90 days and 24% were readmitted. General practice provided follow-up for the majority, with most contacts (*n* = 152/174, 87.4%) attributable to the index admission. A total of 50/551 discharge actions were not completed: mostly medication reconciliation. Seven patients experienced harm directly attributable to uncompleted actions, five required hospital readmissions. Most harms were rated substantial and all were preventable or potentially preventable. Dementia and having a recorded carer increased the odds of error, harm, and readmission. Longer hospital stays were linked to higher error risk and costs. All harms followed emergency admissions.

### Strengths and limitations

Including both medical and surgical admissions in the studied cohort enhanced relevance to routine general practice. Though all practices were within one region, purposive sampling captured variation in list size, setting, deprivation, and demographics. A key strength was the use of detailed EHR data, including free-text consultation notes and document attachments, enabling identification of coded and uncoded errors, linking discharge actions to care use, harms, and readmissions, and providing costing information.

A weakness common to all retrospective record analysis studies is that clinically appropriate decisions to override or defer requests may have gone unrecorded. Though harms were assessed independently using standardised tools, some subjectivity remains. The authors did not extract patient postcodes for confidentiality reasons, so individual-level deprivation could not be assessed. Due to the intensity of manual extraction, general practice contact data were limited to the first six contacts.

### Comparison with existing literature

Compared with the authors’ earlier study^1^ in those aged ≥75 years, error rates showed a significant (53%) reduction (*n* = 23/107 [21.5%] versus *n* = 112/246 [45.5%], *P*<0.0001). However, secondary care action requests declined by 22% (*P*<0.0001) in those aged ≥75 years over this time period (64% in 2023 versus 82% in 2014), likely reflecting British Medical Association guidance to reduce workload transfer to primary care.^20,21^ Harm rates were not significantly different. A 2019 meta-analysis found only three of 70 harm studies were set in primary care;^22^ one dataset^23,24^ reporting a 0.5% per patient harm rate in primary care highlights the elevated risk seen in the current study’s cohort (4.4%). Failure to prescribe laxatives (traditionally considered as having low priority to HCPs) was found to be strongly associated with readmission harms in the current study, an effect previously noted in heart failure cohorts.^24^ With their demonstrable five-fold risk of error, people living with dementia (PLWD) are a priority for intervention after discharge.^25^ A meta-analysis found strong evidence for a relative risk (1.42, 95% CI = 1.21 to 1.66) for admission in PLWD,^26^ and cohorts in England and Wales are projected to rise to 1.2–1.7 million by 2040.^27^

The CQC suggests^6^ that the impact of premature discharge may be seen in primary care post-discharge. The current study found general practice costs to be low; secondary care costs by contrast were high with a strong right skew. Patients in the cohort did have a great deal of post-discharge general practice care; the effect of local hospitals’ discharge practices on this expenditure is yet to be studied. The current investigation of general practice costs is timely given the NHS’s ‘left shift’ agenda.^14,28^ Recent NHS efforts to standardise intermediate care data collection illustrate how complex and variable these secondary care pathways are, and underscore the challenges of understanding how such variation impacts the interface with general practice.^29^

Emerging evidence from the US and Israel suggests that when primary care providers have sufficient time to explain discharge summaries to patients, readmissions can be reduced.^30,31^ To replicate this in the UK, primary care must be resourced, better linked with secondary care,^10^ and provided with targeted tools and interventions. Given the perfect separation of error to emergency admissions in the present modelling, future focus should prioritise these discharges.

### Implications for research and practice

General practices should prioritise timely review of high-risk patients, particularly emergency admissions for those with dementia or a carer. Secondary care readmission risk-prediction models should be adapted for primary care. Targeted follow-up (perhaps artifical intelligence enabled) and dedicated, funded time and space for post-discharge review would enable action, with structured tools, for example, discharge summary templates, action checklists, and coded follow-up prompts, to support consistent processing. Understanding how patient and carer communication and continuity of care influence the success of discharge follow-up in general practice is crucial. Improved linkage across primary, community, and hospital datasets may also support more detailed examination of care transitions in future research. Future interventional research should be particularly mindful of resource limitations in general practice, designed to minimise additional workload, and fit around existing literature on post-discharge multidisciplinary teams in general practice, including the NHS Additional Roles Reimbursement Scheme pharmacy roles.^13^

## Data

This study's bespoke dataset was created as part of the General Practice Management After Transition Events (GP-MATE) study, which explores communication between older patients/their carers and their practices after discharge. The data are not publicly available due to the sensitive nature of the pseudonymised healthcare record extraction. Please contact the corresponding author to enquire about data.
